# Dynamic Noise Reduction with Deep Residual Shrinkage Networks for Online Fault Classification

**DOI:** 10.3390/s22020515

**Published:** 2022-01-10

**Authors:** Alireza Salimy, Imene Mitiche, Philip Boreham, Alan Nesbitt, Gordon Morison

**Affiliations:** 1 School of Computing, Engineering and Built Environment, Glasgow Caledonian University, 70 Cowcaddens Road, Glasgow G4 0BA, UK; alireza.salimy@gcu.ac.uk (A.S.); imene.mitiche@gcu.ac.uk (I.M.); a.nesbitt@gcu.ac.uk (A.N.); 2 Innovation Centre for Online Systems, 7 Townsend Business Park, Bere Regis BH20 7LA, UK; pboreham@doble.com

**Keywords:** shrinkage function, thresholding, EMI method, classification, machine-learning, condition monitoring, de-noising

## Abstract

Fault signals in high-voltage (HV) power plant assets are captured using the electromagnetic interference (EMI) technique. The extracted EMI signals are taken under different conditions, introducing varying noise levels to the signals. The aim of this work is to address the varying noise levels found in captured EMI fault signals, using a deep-residual-shrinkage-network (DRSN) that implements shrinkage methods with learned thresholds to carry out de-noising for classification, along with a time-frequency signal decomposition method for feature engineering of raw time-series signals. The approach will be to train and validate several alternative DRSN architectures with previously expertly labeled EMI fault signals, with architectures then being tested on previously unseen data, the signals used will firstly be de-noised and a controlled amount of noise will be added to the signals at various levels. DRSN architectures are assessed based on their testing accuracy in the varying controlled noise levels. Results show DRSN architectures using the newly proposed residual-shrinkage-building-unit-2 (RSBU-2) to outperform the residual-shrinkage-building-unit-1 (RSBU-1) architectures in low signal-to-noise ratios. The findings show that implementing thresholding methods in noise environments provides attractive results and their methods prove to work well with real-world EMI fault signals, proving them to be sufficient for real-world EMI fault classification and condition monitoring.

## 1. Introduction

Power generation equipment and assets used in high-voltage (HV) power production plants are prone to developing faults; if these faults are undetected they can lead to breakdowns, in turn causing health and safety hazards, legal issues and incurring major losses such as fines and large-scale power outages [1]. Condition monitoring is carried out on HV assets for early fault detection and breakdown prevention, preventing aforementioned losses, and other unwanted outcomes. Condition monitoring is carried out manually by experts [2] observing electromagnetic interference (EMI) data in differing forms based upon individual preferences then following the observations present faults are classified, this method is often used to detect partial-discharge (PD) in assets [3]. The current expert-led manual approach to condition monitoring is problematic operationally due to high cost, sole reliance on experts to detect and classify faults, and lack of continuous monitoring. This leads to faults going unnoticed when experts are not available to carry out condition monitoring practices. An automated approach to condition monitoring will not only reduce dependence on experts but will also allow condition monitoring to be practiced continuously; this will prevent faults from going unnoticed and becoming malfunctions. Reference [4] outlines that the spectrum analysis of specific conditions can lead to more automated methods of data analysis. A continuous automated approach to condition monitoring is implemented using pattern recognition techniques by [5]. With machine learning (ML) also being implemented to focus on condition monitoring using EMI data, [6] implemented a deep residual neural network to classify EMI signals, [7] implements capsule networks to classify EMI signals. Although the produced results from these systems prove to be acceptable, there is room to increase classification results specifically in the presence of interference noise sources. Many of the downfalls of these systems arise from the varying settings found in the data collection process, as these signals are collected to diagnose a variety of assets hence all of the collected signals are subject to different levels of noise.

The work in this study proposes an automated classification system to classify several EMI fault signals in various noise levels. The produced ML system will classify EMI fault signals by using data in the form of Stockwell (S) transform time-frequency (TF) decomposition matrices, introduced by [8], as inputs. The ML system will implement a residual neural network (ResNet), an architecture built for image recognition proposed by [9], alongside various thresholding functions. Thresholding functions, also known as shrinkage functions, are used for signal de-noising; their functionality focuses on thresholding parameters often chosen by signal processing experts, to outline and alter values they believe to be in noise ranges; they then use the thresholding functions and their chosen parameters to create filters. The systems produced in this study implement architectures that learn thresholding parameters through model training, creating an automated de-noising filter. This automation allows de-noising without expert insight into the data being observed. Previous work using the learned thresholding method has been carried out by [10] focusing on the soft thresholding method and applying it to perform condition monitoring on mechanical power generation assets, the work implements learned thresholding by using a ResNet architecture with residual shrinkage blocks. The work in this study will build upon the residual-shrinkage-building-unit from [10] (RSBU) by implementing, to the author’s knowledge, previously unexplored methods of thresholding in the condition monitoring field, building a new residual-building-unit-2 (RSBU-2) architecture to perform thresholding functions that require two thresholding parameters and applying the built methods on EMI data. The EMI fault signal dataset used in this study is collected from real-world operational HV assets, containing 8 fault classes. This data was used to train and test 7 different learned thresholding systems, 4 of which contained a single learned thresholding parameter and the remaining 3 consisted of two learned thresholding parameters.

The following sections of this paper will outline the methods used in this study and will be structured as follows; Section 2 will introduce the S transform, Section 3 describes the thresholding functions implemented throughout the study outlining their learned parameters, Section 4 introduces and explains the two models used in the study, Section 5 outlines the experimental set-up of the research describing the dataset and tools used, Section 6 presents and discusses the research findings outlining how models were evaluated; finally, Section 7 will conclude the findings of the results with insights into future work.

## 2. Stockwell Transform

The feature engineering method used in this study is the S transform, introduced by [8]; this TF distribution is created to build upon the continuous wavelet transform (CWT) while avoiding some of the disadvantages of the method and the S transform is also considered to be a generalisation of the short-time Fourier Transform (STFT).

This study uses the S transform to create TF decompositions of raw time-series EMI signals. The produced TF decomposition retains frequency dependant resolution from the original time-series signals. The retention of this frequency resolution proves to be highly desirable in regards to EMI fault signals from real-world assets due to their non-stationary characteristics. The S transform is selected due to its ability to detect low frequency components of a signal while also providing the ability to detect short lived high frequency components of the signal under analysis, a quality that is not prevalent in the STFT.

### Discrete Stockwell Transform

As the signals used throughout this study are of discrete time nature, the derived TF transform must cohere to this. The discrete time S transform for these signals can be found by performing operations on the Fourier transform, the discrete Fourier transform X[k] of signal x[n] is found using Equation (1).

(1) $$X\left[k\right]=\sum_{n=0}^{N-1}x\left[n\right]e^{-\frac{j2\pi nk}{N}}.$$

The S transform can then be found by carrying out operations on the Fourier transform from Equation (1). This is outlined in Equation (2), where n≠0 and ρ=m=n=0,1,⋯,N−1.

(2) $$S\left[\rho ,n\right]=\sum_{m=0}^{N-1}X\left[m+n\right]e^{-\frac{2\pi^{2}m^{2}}{n^{2}}}e^{\frac{j2\pi m\rho }{N}}.$$

However, Equation (2) does not include the calculation for n=0. To accommodate for this when n=0 is encountered Equation (3) is used.

(3) $$S\left[\rho ,0\right]=\frac{1}{N}\sum_{m=0}^{N-1}x\left[m\right].$$

**Figure 1:**
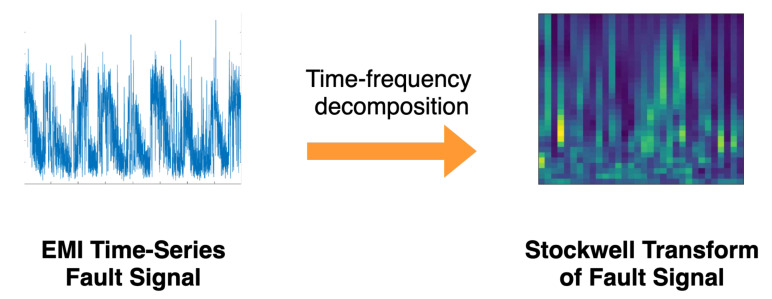
Feature extraction process through the Stockwell transform time-frequency decomposition.

The produced S transform then undergoes further processing to convert the imaginary product of Equation (2) to a modulus product; this is done by taking the absolute value of the S transform |S[ρ,n]|. The feature extraction process through the S transform method can be seen in Figure 1.

## 3. Thresholding Methods

**Figure 2:**
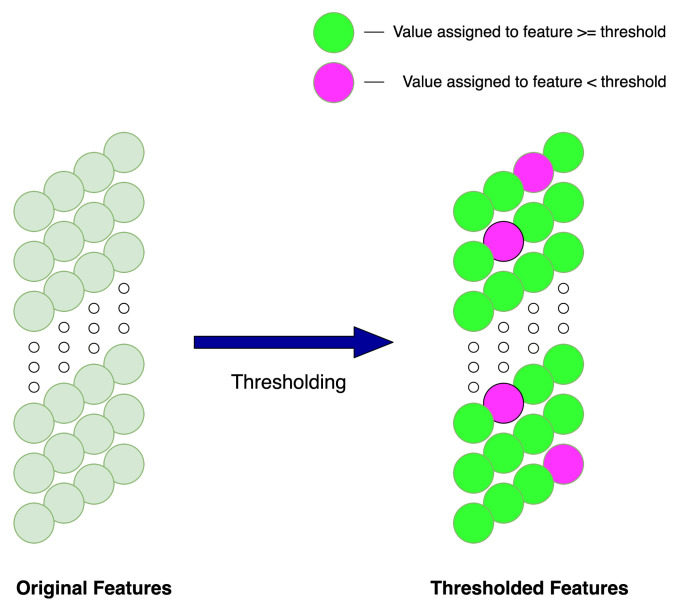
Illustration outlining the feature thresholding method.

Thresholding or shrinkage functions are primarily used for de-noising as observed in [11,12]. The thresholding process consists of creating value ranges which correspond to noise then, dependent upon the thresholding method values both within and outwith these ranges, they are altered. An example of which being the commonly used soft thresholding method from [13]; this thresholding method deems values between the thresholding parameter and zero unimportant, hence all values within this range are set to zero and values outwith the thresholding parameter range are given non-zero values. The thresholding parameter ranges are often chosen by signal processing experts, with expertise in the data at hand. Deep learning reduces dependence on signal processing experts for selecting optimal thresholding parameters for de-noising, and carries out thresholding by learning thresholding parameters through gradient descent, producing thresholding-based filters relevant to the data being observed. Such data-oriented filters should intuitively lead to classification with greater accuracy and allow classifications to occur in high noise environments. The thresholding methods used throughout this study will be used to alter features in the feature map that is undergoing thresholding deemed to correspond to noise; this is outlined in Figure 2.

**Figure 3:**
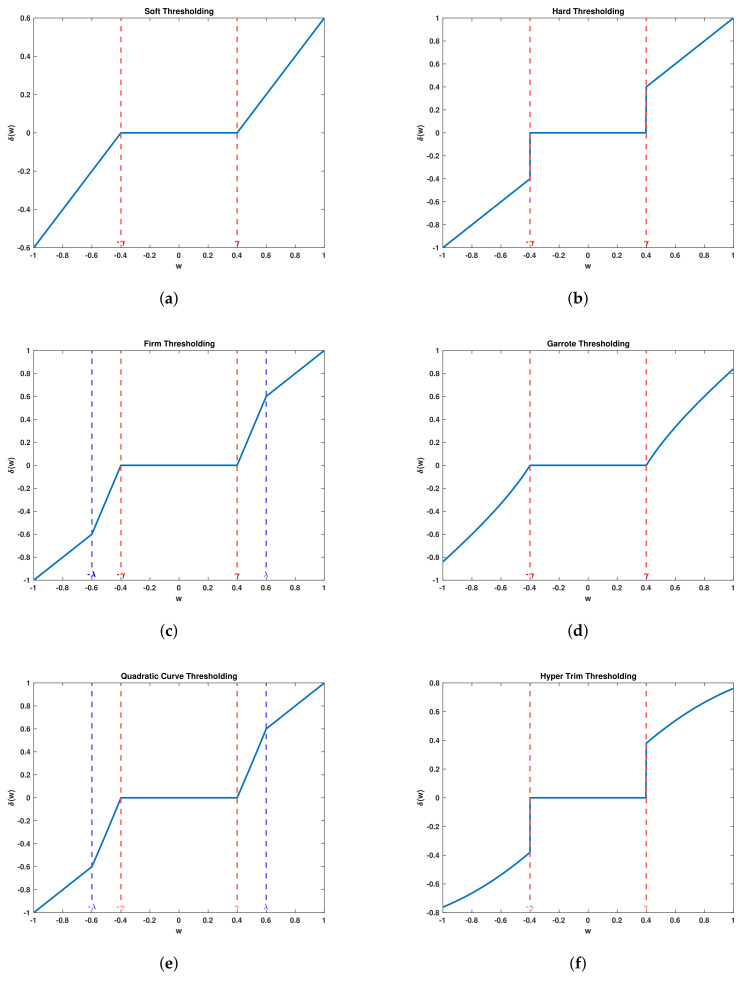
Graphical representation of thresholding methods (**a**)−soft thresholding, (**b**)−hard thresholding, (**c**)−firm thresholding, (**d**)−Garrote thresholding, (**e**)−quadratic curve thresholding, (**f**)−hyper-trim thresholding.

The work carried out in this study will observe several thresholding methods; soft thresholding and hard thresholding [13], firm thresholding [14], Garrote thresholding [15], quadratic curve thresholding (QCT) [16] and Hyper trim (H-trim) thresholding [17]. Thresholding parameters for all of the thresholding methods will be learned using deep learning, graphical representations of the thresholding methods used are found in Figure 3.

### 3.1. Soft Thresholding

The soft thresholding method sets values within the given threshold ranges to zero and values outwith these ranges retain their original values minus the threshold parameter value. This relationship is shown in Equation (4), where γ represents the learned threshold parameter.

(4) $$\delta \left(w\right)=\left\{\begin{array}{cc} 0, & |w|<\gamma \\ (|w|-\gamma )\cdot sgn(w), & |w|\geq \gamma . \end{array}\right.$$

### 3.2. Hard Thresholding

Hard thresholding sets values within threshold ranges to zero and while values outwith the threshold ranges retain their original values; this relationship is shown in Equation (5).

(5) $$\delta \left(w\right)=\left\{\begin{array}{cc} 0, & |w|<\gamma \\ w, & |w|\geq \gamma . \end{array}\right.$$

### 3.3. Garrote Thresholding

Originally introduced to overcome downfalls found in the soft and hard thresholding methods, it has a non-linear approach to values outwith its single thresholding parameter range. Values within the thresholding range are set to zero while values outwith this range are altered non-linearly, as shown in Equation (6).

(6) $$\delta \left(w\right)=\left\{\begin{array}{cc} 0, & |w|<\gamma \\ w-\frac{\gamma^{2}}{w}, & |w|\geq \gamma . \end{array}\right.$$

### 3.4. Firm Thresholding

Firm thresholding consists of two thresholding parameters; this leads to the alteration of values in three ranges. Values within zero and the lower thresholding parameter are set to zero, values within the lower and upper threshold parameters are altered accordingly, and values above the upper threshold limit retain their original values. These relationships are outlined in Equation (7), where γ represents the lower threshold limit and λ represents the upper threshold limit.

(7) $$\delta \left(w\right)=\left\{\begin{array}{cc} 0, & |w|\leq \gamma \\ \frac{\lambda (|w|-\gamma )}{\lambda -\gamma }\cdot sgn\left(w\right), & \gamma \leq |w|<\lambda \\ w, & |w|\geq \lambda . \end{array}\right.$$

### 3.5. Hyper-Trim Thresholding

H-trim thresholding is a method introduced to outperform soft thresholding, prior to the introduction of H-trim, firm thresholding was seen to outperform soft thresholding in electrocardiogram (ECG) signal denoising although firm thresholding had two thresholding parameters compared to H-trims single thresholding parameter. Equation (8) outlines the H-trim thresholding method.

(8) $$\delta \left(w\right)=\left\{\begin{array}{cc} 0, & |w|<\gamma \\ tanh(w), & |w|\geq \gamma . \end{array}\right.$$

### 3.6. Quadratic Curve Thresholding

QCT is a method introduced to overcome the weaknesses found in soft and hard thresholding. Overcoming the Gibbs phenomenon, outlined in [18], caused by an overshoot at jump discontinuities when thresholds are achieved in hard thresholding and distortion found when |w|≥γ in soft thresholding [16]. QCT contains two thresholding parameters and three coefficients, Equation (9) shows the mathematical relationships for QCT. Where γ and λ represent the lower and upper thresholding parameters respectively and the coefficients used are represented by *a*, *b*, and *c*.

(9) $$\delta \left(w\right)=\left\{\begin{array}{cc} 0, & |w|<\gamma \\ aw^{2}+bw+c\gamma , & \gamma \leq |w|<\lambda \\ w, & |w|\geq \lambda . \end{array}\right.$$

Values within the lower threshold range are set to zero, values within the lower and upper threshold range are assigned a quadratic curve value and finally values greater than the upper threshold range retain their original values. Given that (γ,0) and (λ,λ) are points on the same quadratic curve, the set of equations in Equation (10) can be found.

(10) $$\left\{\begin{array}{c} 0=a\gamma^{2}+b\gamma +c\\ \lambda =a\lambda^{2}+b\lambda +c. \end{array}\right.$$

Equation (10) can then be further simplified to describe *b* and *c* with respect to the thresholding ranges, this is shown in Equations (11) and (12) respectively. Showing three parameters to be assigned values, γ and λ, which are the learned thresholding parameters and *a*, which is a coefficient to be assigned a value, a=1 was used for the purposes of this study.

(11) $$b=\frac{\lambda }{\lambda -\gamma }-a\left(\lambda +\gamma \right)$$

(12) $$c=\gamma \lambda [\frac{1}{\gamma -\lambda }+a].$$

## 4. Model Architecture

The studies throughout this work implemented two neural-network architectures based upon the DRSN proposed in [10]. Architectures are developed using a deep ResNet architecture along with RSBUs, ResNet architectures developed in [9] that are a form of convolutional neural network (CNN) that are widely used in the image classification field due to their state-of-the-art performance on image classification tasks. The main alteration in ResNets in comparison to conventional CNNs is the presence of identity skip connections which reduce training error and loss, due to their ability to eliminate the vanishing or exploding gradient problem.

This study builds upon the work in [10] by implementing further thresholding methods, with both single and two thresholding parameters, to tackle EMI signal classification tasks, building upon the DRSN architecture to accommodate thresholding methods with two learned thresholds and observing the benefits of using these alternative forms of learned thresholding methods.

**Figure 4:**
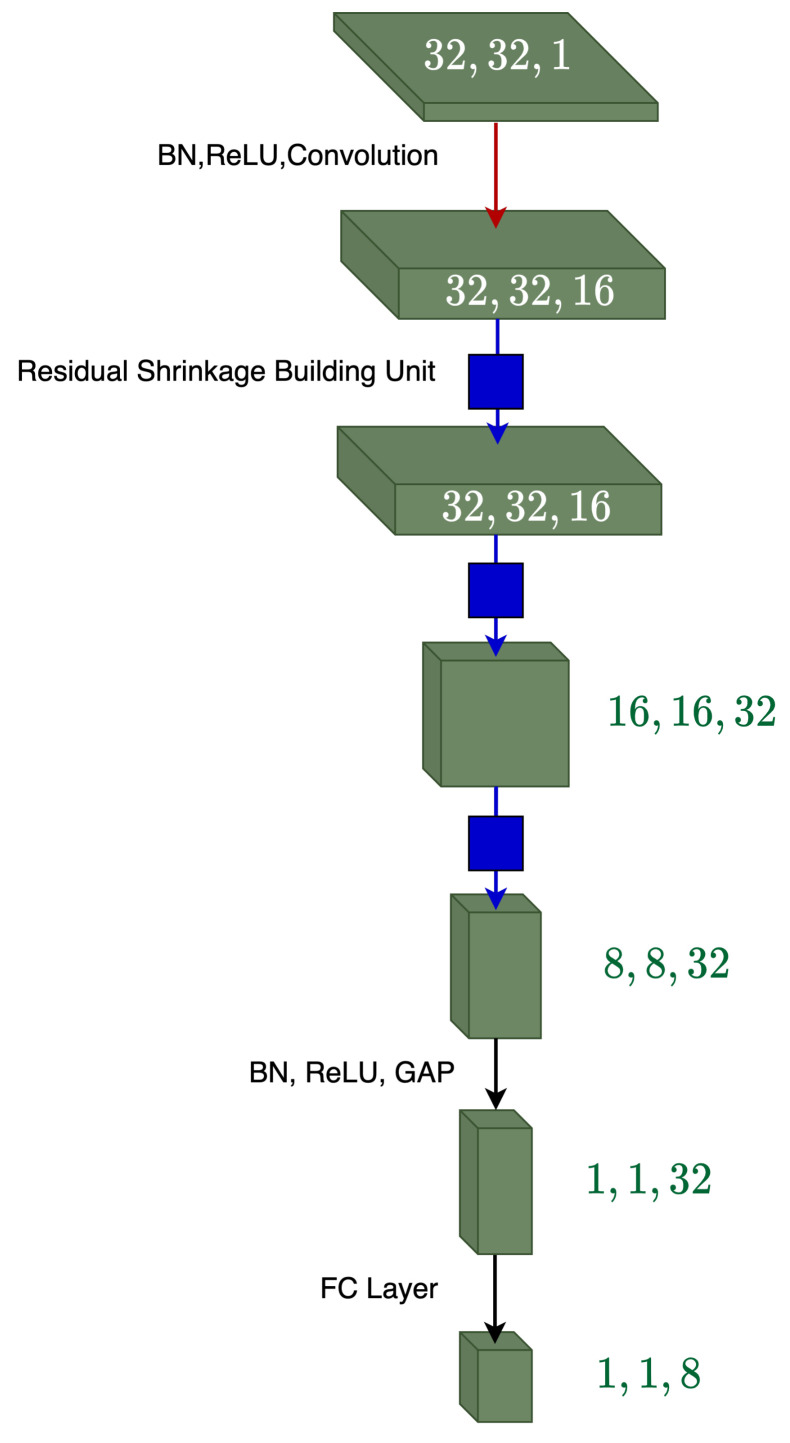
Overall deep residual shrinkage network.

The overall DRSN architecture used in this study can be found in Figure 4, the architecture is shown to stack RSBU blocks to gradually decrease noise-related features. Alterations in the RSBU are proposed for learning two thresholding parameters. This new architecture is referred to as RSBU-2 with the original architecture for learning a single thresholding parameter being referred to as RSBU-1.

### 4.1. RSBU-1

**Figure 5:**
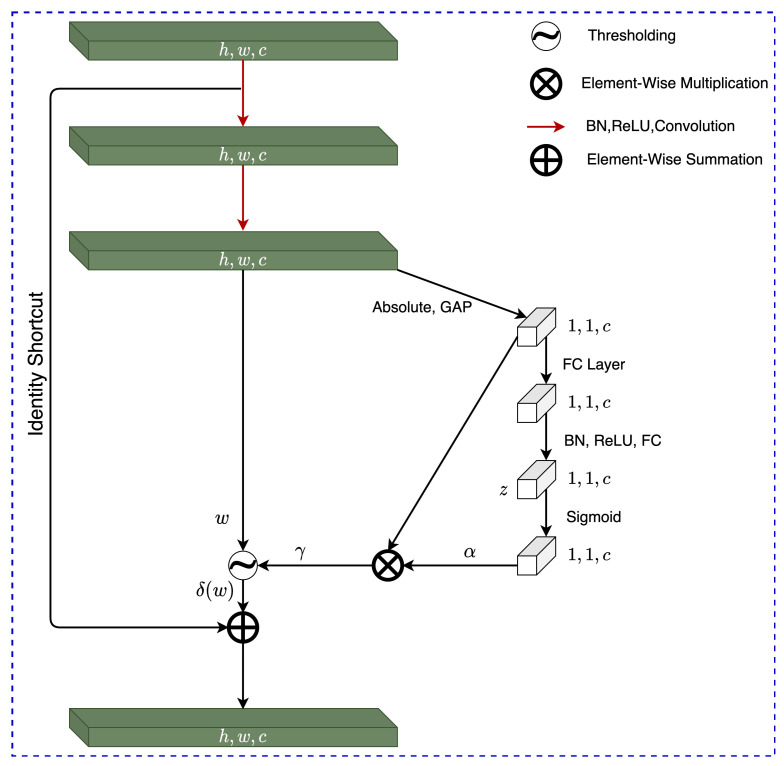
Residual Shrinkage Building Unit-1 Architecture.

Designed to learn a single thresholding parameter, the RSBU-1 architecture can be found in Figure 5. The channel-wise threshold parameter γ(c) is found by following several steps. Firstly, through finding the absolute value of the global average pooling (GAP), the feature map is reduced to a one-dimensional vector. GAP provides many benefits to the overall architecture and the operation calculates the mean value from each channel of the input feature map, in turn reducing the number of weights to be used in the following FC layer reducing the probability of encountering over-fitting. The one-dimensional vector is then propagated to a two-layer fully connected (FC) network and the output of this network can be found in Figure 5 as α(c), which is the scaling parameter of the *c*th neuron and it is scaled to the range (0,1) using Equation (13), where z(c) represents the feature of the *c*th neuron; this relationship is shown as the Sigmoid layer in Figure 5.

(13) $$\alpha \left(c\right)=\frac{1}{1+e^{-z(c)}}.$$

The discovered scaling parameter α(c) is then used to find the thresholding parameter of the *c*th channel of the feature map γ(c) through element-wise multiplication of α(c) and the output of the absolute GAP shown as $\underset{\left(h,w\right)}{E}\left[w\left(h,w,c\right)\right]$, this relationship is found in Equation (14), where *h*, *w* and *c* show the indexes of the height, width and channels of the feature map *w*.

(14) $$\gamma \left(c\right)=\alpha \left(c\right)\cdot \underset{\left(h,w\right)}{E}\left[w\left(h,w,c\right)\right].$$

The RSBU-1 architecture was used to find the single thresholding parameters for soft, hard, Garrote, and H-trim thresholding.

### 4.2. RSBU-2

**Figure 6:**
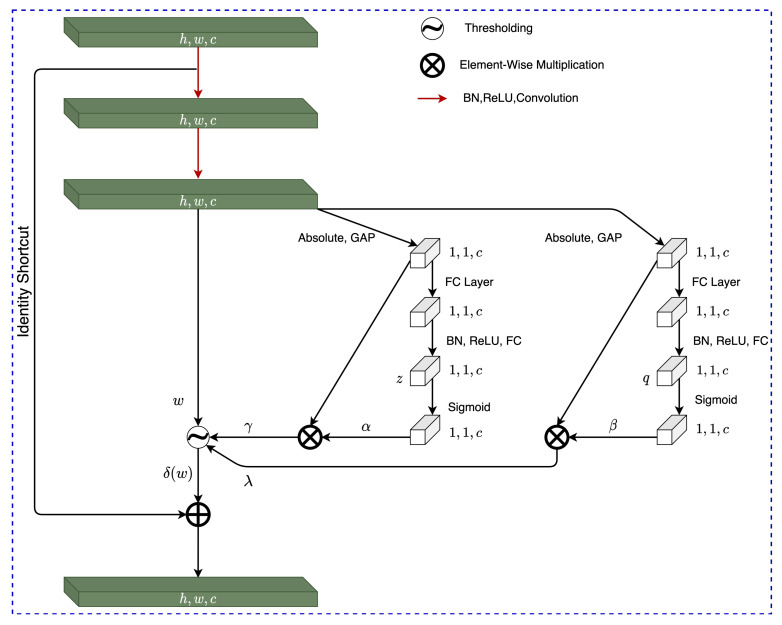
Residual Shrinkage Building Unit-2 Architecture.

The RSBU-2 architecture, found in Figure 6, was created to accommodate a second thresholding parameter referred to in this work as λ. The first channel-wise thresholding parameter γ(c) of RSBU-2 is found identically to the methods explored in RSBU-1 and outlined in Section 4.1. The discovery of the second channel-wise thresholding parameter λ(c) is outlined in this sub-section.

First, the feature map *w* undergoes absolute GAP, the resultant one-dimensional vector is propagated to a two-layer FC network and the produced output of the FC network q(c) is scaled using Equation (15) producing the scaling parameter of the *c*th channel β(c).

(15) $$\beta \left(c\right)=\frac{1}{1+e^{-q(c)}}.$$

λ(c) is then found by carrying out an element-wise multiplication between the calculated β(c) and the absolute GAP of *w* shown as $\underset{\left(h,w\right)}{E}\left[w\left(h,w,c\right)\right]$; this relationship is outlined in Equation (16).

(16) $$\lambda \left(c\right)=\beta \left(c\right)\cdot \underset{\left(h,w\right)}{E}\left[w\left(h,w,c\right)\right].$$

The RSBU-2 architecture was used to find two learned thresholding parameters γ and λ, and was used to implement firm and QCT thresholding.

## 5. Experimental Set-Up

**Figure 7:**
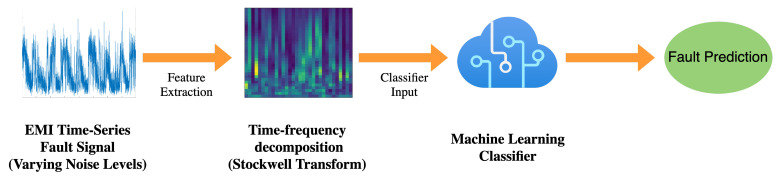
Flow diagram showing the proposed system, outlining feature extraction and classification of EMI signals at varying noise level inputs.

The work in this study used real-world fault signals to produce an automated noise-robust classification model using ML methods, this section will outline the necessary steps taken to process the signal data in preparation for model ingestion outlining the steps taken to produce datasets at various known noise levels, feature engineering, in the form of TF decomposition methods, to create data that can be ingested into the classification models, and how the implemented classification models underwent training, validation, and testing. Figure 7 outlines the feature extraction and classification system used to produce fault predictions from EMI signals at varying noise levels.

### 5.1. Data Set

**Figure 8:**
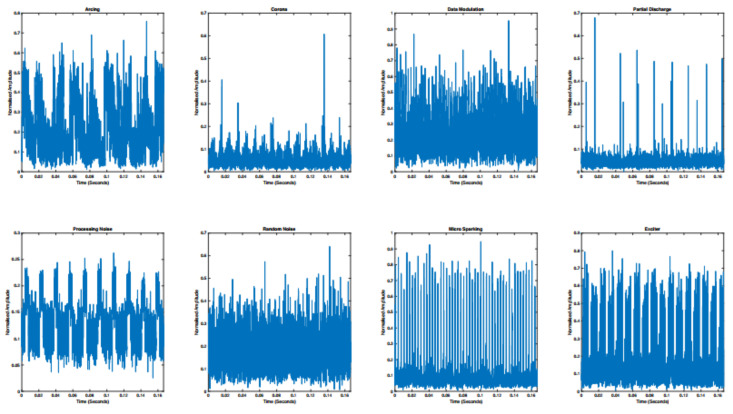
Raw time-series EMI fault signals, representing each prevalent class.

The EMI signals used in this study were collected in adherence with the Comité International Spécial des Pertrubations Radioélectriques (CISPR) 16 standard from [19], and implemented the EMI signal collection technique from [20]. The collected data were in the form of time-resolved signals from real-world operational assets sampled at a rate of 24000 samples/second and signals were labelled with their corresponding faults through manual expert analysis. The raw time series signals can be seen in Figure 8. Eight fault classes were prevalent in the dataset signals; PD, Exciter, Arcing, Data-Modulation, Processing Noise, Random Noise, and Micro-Sparking. A balanced dataset was produced from the collected fault signals and each class was represented with 261 instances with a signal length of 4000 samples per signal.

**Figure 9:**
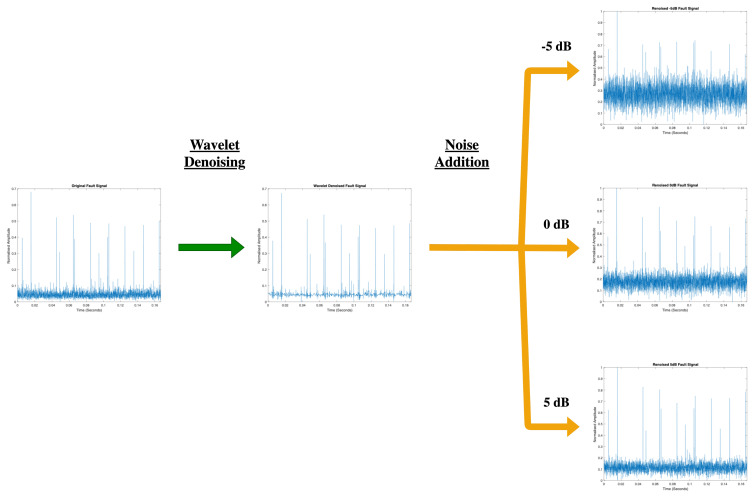
Flow diagram picturing the steps taken to create datasets with varying known noise levels.

The signals in the original dataset then underwent further pre-processing to produce 11 datasets with known noise levels, this was carried out by denoising the original signals using a symlet4 wavelet with a posterior median threshold rule from [21] implemented using the wavelet denoising (wdenoise) method from Matlab and noise variance was estimated based on the highest-resolution wavelet coefficients. Following the denoising of the original signals, additive white Gaussian noise was added at desired levels chosen to be −5, −4, −3, −2, −1, 0, 1, 2, 3, 4 and 5 dB signal-to-noise-ratio (SNR), the process carried out to create the varying noise level datasets is shown in Figure 9. All 11 known noise datasets were also split into further sub-sections to produce training, validation, and testing splits which contained 70%, 15%, and 15% of signals from their original datasets, respectively.

Prior to model ingestion, the signals in the dataset underwent feature engineering to become two-dimensional real-valued TF mappings, this decomposition was performed using the S transform outlined in Section 2 and then taking the modulus of the produced imaginary TF mapping. Following the feature engineering step, the datasets consist of 261 two-dimensional TF mappings for each fault class, along with the relevant expertly assigned fault label.

### 5.2. Model Training & Testing

The studies required the training of six different learned thresholding architectures and two conventional ML methods, a 50-layer ResNet model (ResNet50) and a 16-layer Visual Geometry Group (VGG16) model proposed in [22], along with eleven alternate known noise datasets. Each architecture and dataset pairing was run ten times, this led to 880 training instances. Model training was implemented using Tensorflow [23], models were trained by reducing categorical cross-entropy loss along with a momentum optimiser with the momentum co-efficient set to 0.9, as recommended by [9], over 650 epochs, the learning rate alternated from 0.1 in the first 260 epochs, then 0.01 in the following 260 epochs, and 0.001 in the final 130 epochs. L2 regularization is used in order to increase test accuracy and reduce the effects of over-fitting; as explained in [24], the L2 regularization penalty term co-efficients were set to 0.0001 staying consistent with classical ResNets [9]. The model with the greatest validation accuracy over all of the epochs in the training instance was selected as the model used for testing. Models with the best validation accuracy from all 880 training runs were tested, and average testing accuracies, and the receiver operating characteristic area under the curve (ROC-AUC) scores were procured through grouping architectures with their model and data pairs, each having ten instances. Testing accuracies were calculated using binary accuracy, which is the division of the total number of correct predictions by the number of test samples. Mathematically described in Equation (17), where TP, TN, FP, and FN, represent true positives and negatives and false positives and negatives respectively. The ROC-AUC score is the area under the probability curve, also known as the ROC; the ROC-AUC score is limited between 0→1 and a value closer to 1 shows a model with greater class separability, meaning the model is better at distinguishing between classes.

(17) $$\mathrm{Acc}=\frac{correct\;predictions}{all\;predictions}=\frac{TP+TN}{TP+TN+FP+FN}$$

## 6. Results & Discussion

**Figure 10:**
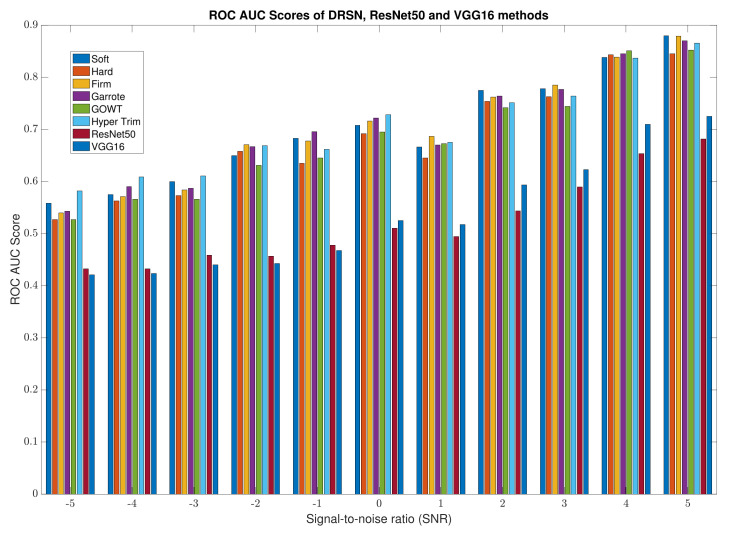
Bar graph of average receiving operating characteristics area under the curve score of alternative thresholding, ResNet50 and VGG16 methods. DRSN with RSBU-1 is used to implement Soft, Hard and Garrote thresholding, DRSN with RSBU-2 is used to implement H-trim, Firm and QCT thresholding.

The mean test accuracies over the ten runs are provided by testing the thresholding architectures against corresponding known noise level data and the results can be found in Table 1. Figure 10 outlines the corresponding ROC-AUC scores of the various thresholding methods, implemented through the proposed DRSN networks, and the conventional ML architectures, with the alternating known noise level datasets. The results show thresholding models with two thresholding parameters to outperform their single thresholding parameter counterparts in high-noise environments when considering mean test accuracies. The results also outline the benefits of exploring further thresholding methods in the HV condition monitoring field, as to the author’s knowledge soft thresholding is the only learned thresholding method used in the condition monitoring field prior to this study and it is shown to be outperformed in a majority of noise environments in EMI signal data. The DRSN networks proposed also show vast performance improvements when compared to state-of-the-art conventional ML classification architectures showing double figure improvements in mean test accuracies in all noise environments, with further support when comparing mean ROC-AUC scores.

## 7. Conclusions

The work in this study produced several thresholding methods to tackle the noisy environments in which HV condition monitoring, through EMI signal analysis, incurs. Providing methods that automate the denoising and classification process of EMI signals, the results of the work found the benefits of exploring novel thresholding methods in the field, how these thresholding methods can be used with ML to produce a non-expert dependant system to carry out denoising prior to classification, and the work produced an architecture to allow for the learning of several thresholding parameters when required for thresholding methods which require more than one learned parameter. The architecture proposed in this work showed significant improvements in current conventional ML classification methods.

The systems produced in this work can be used to develop an adaptable condition monitoring software to reduce dependence on both HV condition monitoring experts and signal processing experts. Creating an adaptable and robust fault classification system will benefit users and producers of electricity by ensuring the prevention of devastating losses. The work from the study can be built upon by observing further thresholding methods and exposing these methods to assets in real-world settings.

## Figures and Tables

**Table 1:** Average Testing accuracies of thresholding, ResNet50 and VGG16 models with alternate noise level data. Best performance is presented in **bold** font. DRSN with RSBU-1 is used to implement Soft, Hard and Garrote thresholding, DRSN with RSBU-2 is used to implement H-trim, Firm and QCT thresholding.

Data Set (dB SNR)	Soft	Hard	Garrote	H-trim	Firm	QCT	ResNet50	VGG16
−5	53.54%	52.63%	53.72%	53.94%	54.71%	**56.09**%	43.21%	42.12%
−4	58.10%	55.51%	57.59%	57.48%	60.07%	**60.84**%	43.25%	42.34%
−3	58.32%	59.74%	60.62%	58.61%	60.15%	**61.68**%	45.84%	43.98%
−2	62.85%	60.18%	63.10%	61.39%	60.88%	**64.42**%	45.69%	44.27%
−1	62.48%	60.29%	61.13%	60.36%	**62.85**%	62.70%	47.78%	46.75%
0	67.12%	67.15%	67.77%	65.77%	**68.50**%	67.59%	51.02%	52.52%
1	75.07%	74.56%	76.06%	73.91%	74.64%	**76.13**%	49.42%	51.75%
2	**79.12**%	77.15%	77.70%	76.02%	77.52%	74.38%	54.34%	59.34%
3	79.31%	76.53%	**80.55**%	77.88%	78.72%	79.20%	58.94%	62.26%
4	85.84%	83.21%	**86.42**%	83.58%	85.00%	85.00%	65.36%	70.99%
5	**84.38**%	80.47%	83.58%	81.57%	81.28%	83.83%	68.14%	72.48%

## Data Availability

The data used in this research is private due to the client confidentiality policy of Doble Engineering Company.

## Abbreviations

The following abbreviations are used in this manuscript:

HV	High-voltage
EMI	Electro-magnetic interference
DRSN	Deep residual shrinkage networks
RSBU	Residual shrinkage building unit
PD	Partial discharge
ML	Machine Learning
S	Stockwell
ResNet	Residual Neural Network
CWT	Continuous wavelet transform
TF	Time-frequency
QCT	Quadratic curve thresholding
ECG	Electrocardiogram
CNN	Convolutional neural network
GAP	Global average pooling
FC	Fully connected
CISPR	Comité International Spécial des Pertrubations Radioélectriques
SNR	Signal-to-noise-ratio
VGG	Visual Geometry Group
